# The Alzheimer's disease‐associated complement receptor 1 variant confers risk by impacting glial phagocytosis

**DOI:** 10.1002/alz.70458

**Published:** 2025-07-09

**Authors:** Nikoleta Daskoulidou, Bethany Shaw, Wioleta Milena Zelek, Bryan Paul Morgan

**Affiliations:** ^1^ UK Dementia Research Institute at Cardiff University, School of Medicine Cardiff University Cardiff UK

**Keywords:** Alzheimer's, astrocytes, complement, CR1, microglia, opsonization, phagocytosis

## Abstract

**INTRODUCTION:**

Genome‐wide association studies have implicated complement in Alzheimer's disease (AD). The CR1*2 variant of complement receptor 1 (CR1; CD35), confers increased AD risk. We confirmed CR1 expression on glial cells; however, how CR1 variants influence AD risk remains unclear.

**METHODS:**

Induced pluripotent stem cell‐derived microglia and astrocytes were generated from donors homozygous for the common CR1 variants (CR1*1/CR1*1;CR1*2/CR1*2). CR1 expression was quantified and phagocytic activity assessed using diverse targets (*Escherichia coli* bioparticles, amyloid β aggregates, and synaptoneurosomes), with or without serum opsonization.

**RESULTS:**

Expression of CR1*1 was significantly higher than CR1*2 on glial lines. Phagocytosis for all targets was markedly enhanced following serum opsonization, attenuated by Factor I‐depletion, demonstrating CR1 requirement for C3b processing. CR1*2‐expressing glia showed significantly enhanced phagocytosis of all opsonized targets compared to CR1*1‐expressing cells.

**DISCUSSION:**

CR1 is critical for glial phagocytosis of opsonized targets. CR1*2, despite lower expression, enhances glial phagocytosis, providing mechanistic explanation of increased AD risk.

**Highlights:**

- Induced pluripotent stem cell (iPSC)‐derived glia from individuals expressing the Alzheimer's disease (AD) risk variant complement receptor (CR) 1*2 exhibit lower CR1 expression compared to those from donors expressing the non‐risk form CR1*1.
- The iPSC‐derived glia from individuals expressing the AD risk variant CR1*2 exhibit enhanced phagocytic activity for opsonized bacterial particles, amyloid‐β aggregates and human synaptoneurosomes compared to those from donors expressing the non‐risk form CR1*1.
- We suggest that expression of the CR1*2 variant confers risk of AD by enhancing the phagocytic capacity of glia for opsonized targets.

## Graphical abstract

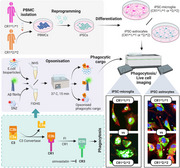

## 1 BACKGROUND

The complement system plays crucial roles in protecting against pathogens and removing debris during the healing process.^1^, ^2^ When dysregulated, complement can cause uncontrolled inflammation and tissue damage, observed in diverse disease contexts. In the case of Alzheimer's disease (AD), complement activation, inflammation, and glial cell activation have long been recognized as features of the disease, albeit considered secondary to amyloid‐β (Aβ) and tau‐related pathology;^3^ however, recent genetic studies suggest that complement plays a primary role in AD. Notably, *CR1*, which encodes complement receptor 1 (CR1/CD35), and *CLU*, encoding the plasma complement inhibitor clusterin,^4^, ^5^ are major hits in AD genome‐wide association studies (GWAS), while *CFH*, the gene encoding coding for the complement regulator factor H (FH), has also been implicated.^6^, ^7^ Other GWAS findings include *CD33* and *TREM2*, both of which encode myeloid cell receptors that bind complement proteins C1q and/or C3.^8^, ^9^ The precise mechanisms by which complement gene variants influence the risk of AD are still unknown. Understanding the expression patterns of the implicated genes and their impact on brain homeostasis would enhance our comprehension of AD pathogenesis and potentially lead to improved diagnostic and therapeutic approaches targeting the complement system in AD.

**FIGURE 1 alz70458-fig-0001:**
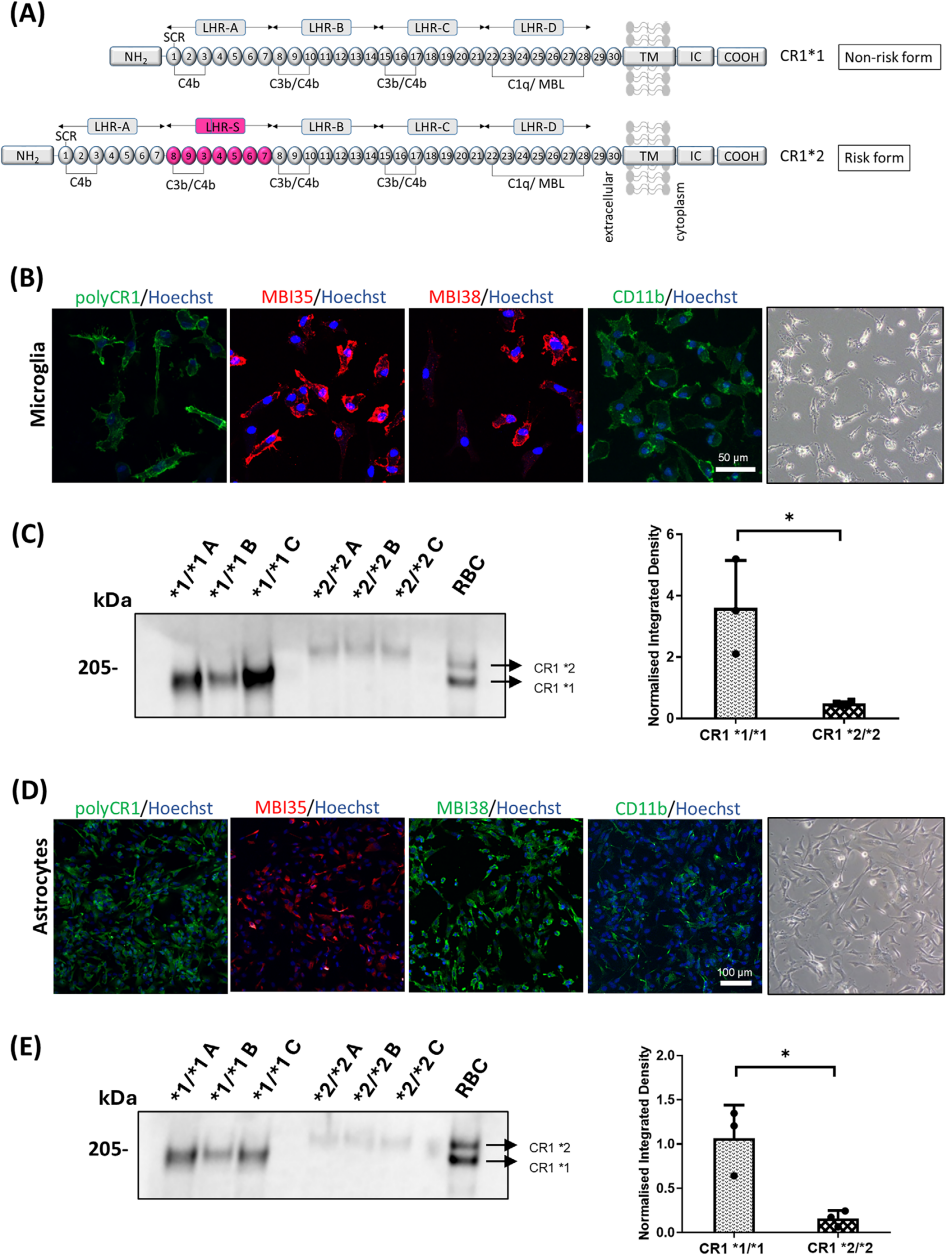
Complement receptor 1 (CR1) expression in CR1*1/*1 versus CR1*2/*2 induced pluripotent stem cell (iPSC) ‐microglia and astrocytes. (A) Protein domains of common CR1 variants. CR1*1, the most common form of CR1, comprises four long homologous repeats (LHRs) each composed of seven short consensus repeats (SCRs) of 60–70 amino acids. There are three C4b binding sites (SCR 1‐3, 8‐10, and 15‐17) and two C3b binding sites (SCR 8‐10 and 15‐17). SCRs 22‐28 bind C1q, MBL, and ficolins. CR1*2, the Alzheimer's disease (AD)‐associated variant, has an additional LHR domain (LHR‐S) and consequently an extra C3b/C4b binding site. NH_2_: amino terminus; TM: transmembrane segment; IC‐COOH: intracytoplasmic carboxy‐terminal domain. Adapted from Ref. 13. (B) Immunofluorescence staining of CR1 in iPSC‐microglia, showing membrane‐associated labeling with a polyclonal antibody (pAb) anti‐CR1 and monoclonal antibodies (mAbs) MBI38 and MBI35. CD11b, a phagocytic receptor, is also shown. Maximum projections of Z‐stacks are presented, with representative images of CR1*1/CR1*1 and CR1*2/CR1*2 homozygous lines. (C) Western blot analysis of CR1 expression in CR1*1 and CR1*2 iPSC‐microglia, using mAb 3E10, which recognizes a single epitope on CR1. Densitometric analysis (right) shows CR1 expression levels normalized to total protein from stained gels, comparing CR1*1‐ and CR1*2‐expressing cells. Red blood cell (RBC) lysate from a CR1*1/CR1*2 donor was used as a positive control. (D) CR1 expression in iPSC‐astrocytes, as described in (B). (E) Western blot analysis of CR1 expression in CR1*1 and CR1*2 iPSC‐astrocytes, as in (C). Although CR1 expression is notably higher in microglia compared to astrocytes, this reflects differences in protein expression levels rather than differential reactivity of the antibody between the two cell types. *p < 0.05.

In this study, we focus on investigating the GWAS hit *CR1*; the gene encodes a membrane receptor for the C3 activation product C3b, which plays crucial roles in regulating complement activation and phagocytosis. It achieves this by accelerating the decay of the C3 convertase and acting as a cofactor for factor I (FI)‐mediated conversion of C3b to iC3b. The latter serves as a ligand for the important integrin phagocytosis receptors complement receptor 3 (CR3; CD11b/CD18) and complement receptor 4 (CR4; CD11c/CD18).^10^ The CR1 gene, located in the “regulators of complement activation” gene cluster on chromosome 1q32, encodes a type 1 transmembrane protein comprising a chain of repeating units, each ∼60 amino acids long, called short consensus repeats (SCRs). These SCR units are organized into sets of seven, known as long homologous repeats (LHRs), with each LHR a distinct functional unit with unique complement binding properties (Figure 1A). There are four codominant CR1 alleles differing in terms of LHR number; the commonest, CR1*1 (allele frequency 0.87), comprises four LHRs, while the CR1*2 variant (allele frequency 0.11) includes an additional LHR (LHR‐S) inserted between LHRs A and B, thereby providing an extra binding site for C3b/C4b.^11^ Notably, CR1*2 is strongly associated with risk of late‐onset AD, faster cognitive decline, and greater neuropathological burden.^12^, ^13^ Counterintuitively, CR1*2 expression is associated with lower levels of brain Aβ;^14^ this observation provoked the suggestion that Aβ might be redistributed into a more neurotoxic form in the presence of CR1*2.^15^

The cellular and molecular roles of CR1 in the brain and how it influences AD risk are poorly understood. Even the expression of CR1 in the brain was a subject of controversy, recently resolved by us.^16^ We took a multipronged approach to test CR1 expression in human microglia in situ and in vitro in induced pluripotent stem cell (iPSC)‐derived microglia expressing the risk (CR1*2) or non‐risk (CR1*1) variants and human brain tissue; CR1 transcript and protein were expressed in iPSC‐microglia in vitro and on microglia and astrocytes in situ in human brain. The observed expression of CR1 on immune cells in the brain supports a direct role of CR1 in immune and inflammatory processes in the heathy and diseased brain. Here we extended these findings by investigating impact of expression of CR1 variants on microglial and astrocytic phagocytic functions.

## 2 METHODS

### 2.1 Reagents and plastics

All reagents and tissue culture plastics, except where otherwise stated, were from Fisher Scientific (Loughborough, UK) or Sigma Aldrich (Gillingham, UK) and are of analytical grade.

### 2.2 Human tissue

Frozen *post mortem* brain tissue (region BA41/42) from five AD cases (Braak VI) and five age‐ and sex‐matched controls were used (Table S1) for isolation of synaptoneurosomes and one of each was chosen for phagocytosis. Blood samples were collected from consented healthy donors and anonymized. Erythrocyte membranes were isolated by standard methods.

### 2.3 Synaptoneurosome isolation and labeling

Synaptoneurosome (SNZ) isolation followed established protocols.^17^ Protein concentrations at all stages were measured using the Pierce Micro BCA (bicinchoninic acid) Protein Assay Kit (ThermoFisher). Snap‐frozen human brain tissue (300–500 mg from superior temporal gyrus BA41/42) was homogenized on ice in a Dounce homogenizer, suspended in 1 mL ice‐cold Buffer A [25 mM HEPES, 120 mM NaCl, 5 mM KCl, 1 mM MgCl_2_, 2 mM CaCl_2_, pH 7.5, supplemented with protease and phosphatase inhibitors (Merck, 11836170001; 524629‐1SET)] then passed through an 80 µM filter (Merck, NY8002500) to obtain total brain homogenate (TBH). An aliquot of TBH was retained, stored at −80°C, the remainder passed through a 5 µM filter (Merck, SLSV025LS) to remove cells, large organelles and nuclei, a portion retained for Western blot validation and the remainder centrifuged at 1000 x g for 5 min. The resultant SNZ pellet was washed with Buffer A and either snap‐frozen in aliquots and stored at −80°C or dye‐labeled. For labeling SNZ, we used a published method;^18^ SNZ were resuspended to 2 mg/mL in 100 mM NaHCO_3_ buffer pH 9, incubated with pHrodo Red‐SE (ThermoFisher, P36600; stock 10 mM in dimethyl sulfoxide [DMSO]) at 50 µM with shaking in the dark for 1 h at room temperature, centrifuged (13,000 x g, 10 min) to pellet the labeled SNZ, washed, resuspended in 1 mL in phosphate buffered saline (PBS)/5% DMSO to minimize ice crystal formation, prevent aggregation, and preserve the dye, then aliquoted and frozen at −80°C at ∼5 mg protein/mL.

### 2.4 Preparation of depleted sera

FI was depleted from human serum by passage at 4°C over a 5 mL HiTrap NHS‐Activated HP column (Cytiva) on which 15 mg of OX21 anti‐FI monoclonal antibody (mAb, ECACC #060417) was immobilized. Serum fractions were collected on ice and FI depletion confirmed by enzyme‐linked immunosorbent assay (ELISA) and Western blotting prior to storage in aliquots at −80°C.

C5 was depleted from serum or FI‐depleted serum by passage over an affinity column as above but with Sky59 mAb anti‐C5 immobilized.^19^ Serum fractions were collected on ice and C5 depletion confirmed by ELISA, Western blotting and hemolysis assay prior to storage in aliquots at −80°C.

**RESEARCH‐IN‐CONTEXT**

1. **Systematic review**: We surveyed relevant literature via PubMed and Google Scholar, focusing on the role of complement receptor 1 (CR1) in the brain. CR1 is expressed in the central nervous system, and the CR1*2 isoform has been consistently identified as a significant genetic risk factor for Alzheimer's disease (AD). Despite this association, the functional consequences of CR1*2 in the context of AD pathogenesis remain poorly defined.
2. **Interpretation**: Our findings underscore the critical role of CR1 in modulating phagocytic function in both microglia and astrocytes. CR1 facilitates the conversion of C3b to iC3b on opsonized substrates, thereby promoting recognition by downstream phagocytic receptors CR3 and CR4. Disruption of this conversion step impairs phagocytosis, confirming a key role for CR1. The CR1*2 risk variant, despite lower surface expression, confers increased phagocytosis of opsonized targets likely via enhanced iC3b generation and interaction with CR3/4.
3. **Future directions**: Although these results offer important mechanistic clues, the link between enhanced opsonic phagocytosis and AD risk remains unresolved. Further studies are needed to delineate how enhanced glial phagocytic capacity contributes to neurodegeneration, with the potential to uncover novel therapeutic strategies aimed at fine‐tuning glial phagocytic activity in Alzheimer's and related disorders.

### 2.5 iPSC culture and differentiation to microglia and astrocytes

Donor‐derived iPSC lines expressing CR1*1 or CR1*2 were generated and fully characterized as previously described.^16^ iPSCs were cultured in mTeSR medium (STEMCELL Technologies) on plates coated with Geltrex LDEV‐free basement membrane matrix (ThermoFisher). All cells were maintained at 37°C, 95% air, and 5% CO_2_, and seeded on black optically clear flat‐bottom tissue culture‐treated 384‐well PhenoPlates (PerkinElmer) or black μclear CELLSTAR tissue culture‐treated 96‐well plates (Greiner Bio‐One Ltd) for live imaging experiments and immunocytochemistry.[Fig alz70458-fig-0001]

iPSC clones from five independent donors were selected for differentiation: one clone from each of the three homozygous CR1*1 donors (*n* = 3) and three clones from the two homozygous CR1*2 donors (*n* = 3), with one donor contributing two clones because of the limited availability of homozygous CR1*2 donors (Table S2). These iPSC clones were differentiated into microglia using the embryoid body (EB) protocol as previously described,^16^, ^20^ or into astrocytes using the STEMdiff Astrocyte differentiation kit and the STEMdiff Astrocyte maturation kit using the manufacturer's EB protocol (STEMCELL Technologies).

### 2.6 Immunofluorescence, SDS‐PAGE, and Western blotting

iPSC‐microglia and astrocytes grown on black optically clear flat‐bottom tissue culture‐treated 384‐well PhenoPlates (PerkinElmer) or black μclear CELLSTAR tissue culture‐treated 96‐well plates (Greiner Bio‐One Ltd) were fixed (4% paraformaldehyde), washed in PBS, incubated in blocking buffer [PBS, 3% bovine serum albumin (BSA), 2% normal goat serum; plus 0.1% Triton X‐100 for intracellular staining], then with primary antibodies at optimal dilutions in blocking buffer overnight. Primary mAb and polyclonal antibody (pAb) against astrocytes marker were anti‐glial fibrillary acidic protein (GFAP, 1:500, rabbit pAb, Abcam #ab7260), anti‐glutamine synthetase (1:200, rabbit pAb, Abcam #ab73593), anti‐excitatory amino acid transporter 1 (EAAT1, 1:100, rabbit mAb, Abcam #ab181036), and anti‐vimentin (1:100, mouse mAb, Abcam #ab8978). Primary antibodies against CD11b (1:100, rat mAb, Abcam #ab8878) and clusterin (in‐house, 4C7; 2 µg/mL) were also used. Primary antibodies against CR1 were: affinity‐purified rabbit pAb anti‐CR1 immunoglobulin (Ig) G (in‐house; purified on immobilized soluble CR1, sCR1); mouse mAb 3E10 generated by immunization with recombinant CR1 SCRs 1–3;^21^ mouse mAb, MBI35 and MBI38 generated in‐house using full‐length sCR1 as immunogen and selected for high affinity binding of CR1. Specificity for all anti‐CR1 reagents was confirmed by demonstrating that preincubation with excess full‐length sCR1 ablated staining and by confirming that CR1 knock‐out lines were negative as described.^16^ Cells were washed, incubated with Alexa fluor conjugated goat anti‐rabbit or anti‐mouse IgG (ThermoFisher) and Hoechst dye, washed, and stored in PBS. Fluorescence was imaged by laser scanning confocal microscopy (Leica SP8); whole‐cell Z‐stacks were assembled for maximum projections. For double staining, primary antibodies were applied together, detected using appropriately labeled secondary antibodies, and imaged using appropriate filters.

For sodium dodecyl sulfate‐polyacrylamide gel electrophoresis (SDS‐PAGE) and Western blotting, cells were lysed in RIPA buffer (ThermoFisher), and protein concentrations were determined using the Pierce BCA Protein Assay Kit (ThermoFisher). Protein (30–40 µg) was diluted in 5× SDS sample buffer (250 mM Tris‐HCl, pH 6.8; 50% (v/v) glycerol; 5% (w/v) SDS; 0.1% (w/v) bromophenol blue), heated at 95°C for 1 min, then resolved on either 7% SDS‐PAGE or 3%–8% tris‐acetate gels (ThermoFisher) alongside PageRuler Plus prestained protein standards (10–250 kDa, ThermoFisher) and transferred onto nitrocellulose membranes. Membranes were blocked using 3% (w/v) skimmed milk powder (Sigma) in PBS containing 0.2% Tween‐20 (PBST) for 1 h at room temperature, then incubated with 4 µg/mL primary mAb 3E10 against CR1 SCR1‐3, which detects a single epitope in either variant,^21^ in blocking buffer overnight at 4°C. After washing with PBST, membranes were incubated with horseradish peroxidase (HRP)‐conjugated secondary antibody (1:10,000 in PBST; Jackson Laboratories) for 1 h at room temperature. Protein bands were detected using enhanced chemiluminescence (ECL) detection reagent (Cytiva) and imaged using the G:BOX imaging system (Syngene). For total protein visualization, gels were stained with GelCode Blue Safe Protein Stain (ThermoFisher) following the manufacturer's instructions. Densitometric analysis was performed using ImageJ (National Institutes of Health), with CR1 expression normalized to total protein levels from stained gels.

### 2.7 Preparation of phagocytosis cargos

pHrodo Red *E. coli* BioParticles (Invitrogen) were reconstituted in live cell imaging solution (LCI; ThermoFisher) at 1 mg/mL, vortexed and sonicated (15 min; Bioruptor sonicator, Diagenode) to disrupt aggregates, diluted to 10 µg/mL in LCI, then incubated with normal human serum (NHS, 1:10 in LCI) to coat with C3b and iC3b or FI‐depleted human serum (FIDHS, 1:10 in LCI) to coat with C3b alone or LCI only for the non‐opsonized group for 30 min at 37°C. Opsonized or non‐opsonized bioparticles were spun down and resuspended in the appropriate amount of LCI before being fed to the cells.

Human HiLyte Fluor 488‐labeled beta‐Amyloid (1‐42) peptides (Anaspec AS‐60479‐01) were reconstituted in 1% NH_4_OH in PBS to 0.1 mM, a concentration reported to induce aggregation,^22^ and stored at −20°C. For opsonization, amyloid aggregates were diluted to 0.5 µM in LCI, then incubated with NHS or FIDHS (1:10 in LCI) for 30 min at 37°C. Opsonized or non‐opsonized amyloid aggregates were resuspended in the appropriate amount of LCI before being fed to the cells.

SNZ tagged with pHrodo prepared as above were resuspended in LCI to 1 mg/mL. For opsonization in the absence of complement lysis, SNZ were incubated with C5‐depleted serum (C5DHS, 1:10 in LCI) or FI/C5‐depleted human serum (FIC5DHS, 1:10 in LCI) for 30 min at 37°C, washed by centrifugation and resuspended in LCI. Opsonized or non‐opsonized SNZ were fed to the cells at 1 µg/well.[Fig alz70458-fig-0002]

### 2.8 Flow cytometry of opsonized pHrodo Red *E. coli* BioParticles

Bioparticles were opsonized as described above with NHS or FIDHS or C3‐depleted human serum (C3DHS). To measure opsonization with iC3b, the bioparticles were incubated on ice for 30 min with anti‐human iC3b (10 µg/ml, mouse mAb, Quidel #A290) or isotype control antibody (purified mouse IgG2b,κ, 10 µg/mL, Biolegend #400301), washed in cold LCI solution, and incubated on ice for 15 min with a secondary goat anti‐mouse IgG (H+L) AlexaFluor488 (1:500, Invitrogen #A‐11001). Following another wash in cold LCI, the bioparticles were analyzed on an Attune NxT flow cytometer (ThermoFisher). Bioparticle populations were selected and analyzed using FlowJo software v10.10.00 (BD Biosciences). Negative (no primary) controls, isotype controls and non‐opsonized bioparticles controls were included.

### 2.9 Phagocytosis assays

Myeloid precursors were seeded at a density of 7 × 10^3^ cells per well (50 µL/well) in microglia differentiation media in black optical 384‐well plates (PerkinElmer) and allowed to differentiate into microglia over a period of 10 days. The iPSC‐derived astrocytes cultured in STEMdiff Astrocyte Maturation medium (STEMCELL Technologies) for 20–30 days were seeded at a density of 7 × 10^3^ cells per well (50 µL/well). On the day of the phagocytosis assay, some wells were treated with the cytoskeleton disruptor Cytochalasin D (CytoD; 10 µM, Sigma) for 1 h to disable phagocytosis (negative controls). Cells were washed with 100 µL LCI per well followed by incubation with 25 µL dye mix per well (30 min, 37°C, 5% CO_2_). Dye mix comprised CellTracker Deep Red plasma membrane stain (ThermoFisher, #C34565; 2 mM stock in DMSO diluted to 2 µM in LCI) and 1 drop of NucBlue Live Ready Probes Reagent (Molecular Probes, #R37605) per mL of solution. Plates were washed in 100 µL LCI per well, then transferred to the prewarmed Opera Phenix High‐Content Screening System (PerkinElmer).

For live imaging, 10 fields were captured every 20–30 min over 4–5 h (9–11 time points (T)) for microglia and every 1–2 h over 35‐48 h for astrocytes with a 20x water objective, capturing DAPI (4ʹ,6‐diamidino‐2‐phenylindole; nuclei), Alexa647 (cell tracker), and either Alexa568 (pHrodo) or Alexa488 (for Aβ aggregates) depending on the phagocytic cargo. A T‐1 time point was employed to capture cells prior to the addition of the phagocytic cargo; unmodified or opsonized phagocytic cargo ± CytoD was added and continuous live imaging scanning used for all time points. In some experiments, cells were incubated with simvastatin (Merck, #S6196; 50 µM) to block CR3 to confirm iC3b/CR3‐mediated phagocytosis. At the conclusion of the experiment, the in‐built Harmony analysis software was utilized to quantify phagocytic cargo uptake over time. For each experiment, we measured the average of the three independent iPSC lines per genotype, measured within a single 384‐well plate, a format that permitted testing of all lines and conditions in parallel under identical experimental conditions. Each phagocytosis assay was independently repeated at least three times for each cargo type, demonstrating consistent results. To further ensure reproducibility, microglial assays were performed using three separate differentiations, and astrocyte assays were conducted using two independent batches.

### 2.10 Statistics

All values were expressed as mean ± SEM. Data were plotted using GraphPad Prism v10.04.0 (La Jolla, CA) and tested for normality using the Shapiro–Wilktest (alpha = 0.05). For phagocytosis studies, 3 CR1*1/*1 lines (from 3 donors) and 3 CR1*2/2 lines (from two donors) were examined with at least 12 wells in three independent experiments. All data are presented as the mean ± SEM of three independent lines per genotype and were analyzed using two‐way analysis of variance (ANOVA) followed by Tukey's multiple comparisons test. For Western blot densitometries, data were analyzed using an unpaired *t*‐test. *p < 0.05, **p < 0.01, ***p < 0.001, and ****p < 0.0001.

## 3 RESULTS

### 3.1 CR1 variant expression levels in iPSC‐derived microglia and astrocytes

Three CR1*1/*1 and three CR1*2/2 iPSC lines were selected for differentiation into microglia via EB formation.^16^, ^20^ Differentiation was confirmed by the acquisition of expression of markers of microglia and other myeloid cells (CX3CR1, IBA1, CD45, CD68, and CD11b) and the microglia‐specific marker TMEM119. Surface expression of CR1 was demonstrated on iPSC‐microglia by staining with anti‐CR1 mAbs and pAb (Figure 1B); specificity of antibody staining was previously demonstrated by showing ablation of staining after preadsorption with CR1 protein and absence of expression in knock‐out lines.^16^ CR1 expression was confirmed by Western blotting of lysates from CR1*1 and CR1*2 iPSC‐microglia; for quantification mAb 3E10, specific for SCRs 1‐3 and detecting a single epitope on both CR1 variants was selected,^21^ expression was quantified using ImageJ and normalized to total protein (Figure 1C; Figure S2A). Significantly higher expression of CR1 was observed in CR1*1‐expressing microglia compared to CR1*2.[Fig alz70458-fig-0003]

For differentiation into astrocytes via EB formation (Figure S1A), three CR1*1/*1 and three CR1*2/2 iPSC lines were selected; differentiation was demonstrated by expression of astrocyte marker GFAP, EAAT1, glutamine synthetase, and vimentin (Figure S1B). Surface expression of CR1 was confirmed on iPSC‐astrocytes by staining with anti‐CR1 mAbs and pAb (Figure 1D). CR1 expression was also confirmed by Western blotting of lysates from CR1*1 and CR1*2 iPSC‐astrocytes; quantification was done as for microglia (Figure 1E; Figure S2B). Significantly higher expression of CR1 was observed in CR1*1‐expressing astrocytes compared to the CR1*2 expressing astrocytes.

### 3.2 Microglia and astrocytes expressing CR1*2 show increased phagocytic capacity for opsonized targets

**FIGURE 2 alz70458-fig-0002:**
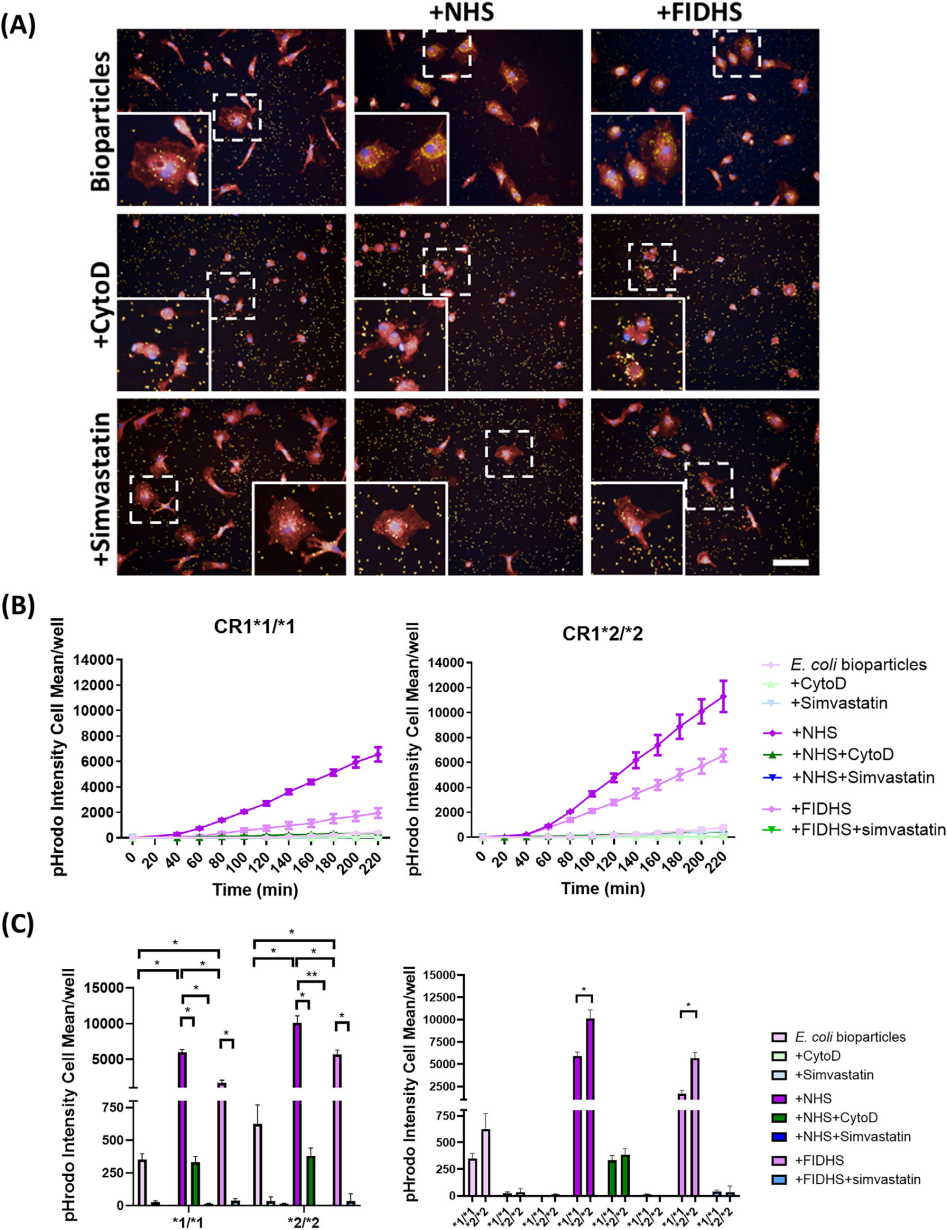
Induced pluripotent stem cell (iPSC) ‐microglia phagocytosis of pHrodo Red *E. coli* BioParticles. Phagocytic cargo was opsonized with normal human serum (NHS) or factor I (FI) ‐depleted human serum (FIDHS). Cytochalasin D (CytoD) was used as negative control. (A) Example pictures showing the cells at 2 h after phagocytosis (pHrodo‐tagged bioparticles: yellow; nuclei: blue; cell tracker: red). Scale bar: 100 µm. (B) Uptake in real time and (C) comparisons at 4 h of phagocytosis. iPSC‐microglia expressing risk variant of complement receptor 1 (CR1) display increased phagocytosis of pHrodo Red *E. coli* bioparticles when compared with non‐risk variant cells. pHrodo Intensity cell mean describes the amount of bioparticle cellular dye emission at each time point. Results are averages of the three independent iPSC lines per genotype, measured within a single 384‐well plate experiment. The phagocytosis assay was independently repeated at least three times demonstrating consistent results. *p < 0.05, **p < 0.01.

To assess the phagocytic capacity of the different iPSC‐microglia lines we first employed pHrodo‐labeled bioparticles derived from *E. coli*, non‐opsonized or opsonized by incubation with NHS or FIDHS, the latter to eliminate cleavage of C3b to iC3b, the ligand for CR3/CR4 (see graphical abstract). Opsonization of bioparticles with iC3b following incubation with NHS was confirmed by flow cytometry (Figure S3). In contrast, iC3b was not detected on bioparticles opsonized with FIDHS or C3DHS, nor on non‐opsonized bioparticles. Isotype and primary‐only controls were included and tested negative for iC3b detection. For all the selected lines, real‐time recording demonstrated slow phagocytic uptake of non‐opsonized *E. coli* bioparticles that was ablated by disruption of phagocytic machinery using CytoD (Figure 2A,B). Opsonization with NHS markedly increased the rate of uptake, while opsonization with FIDHS increased uptake compared to non‐opsonized, though much slower than the NHS‐opsonized group (Figure 2B). Quantification at specific time points confirmed significant enhancement of phagocytosis for NHS‐opsonized compared to either non‐opsonized or FIDHS‐opsonized bioparticles for all the selected lines (Figure 2C). Comparison of iPSC‐microglia expressing the CR1*2 risk variant with those expressing the CR1*1 variant revealed significantly greater phagocytosis of NHS‐opsonized *E. coli* bioparticles in lines expressing the risk variant (Figure 2C). In contrast, there were no significant differences between CR1*1‐expressing and CR1*2‐expressing lines in rates of phagocytosis of non‐opsonized bioparticles. Addition of simvastatin, a well‐characterized CR3 antagonist,^23^ markedly reduced phagocytosis of NHS‐opsonized *E. coli* bioparticles but also reduced uptake of FIDHS‐opsonized bioparticles.

**FIGURE 3 alz70458-fig-0003:**
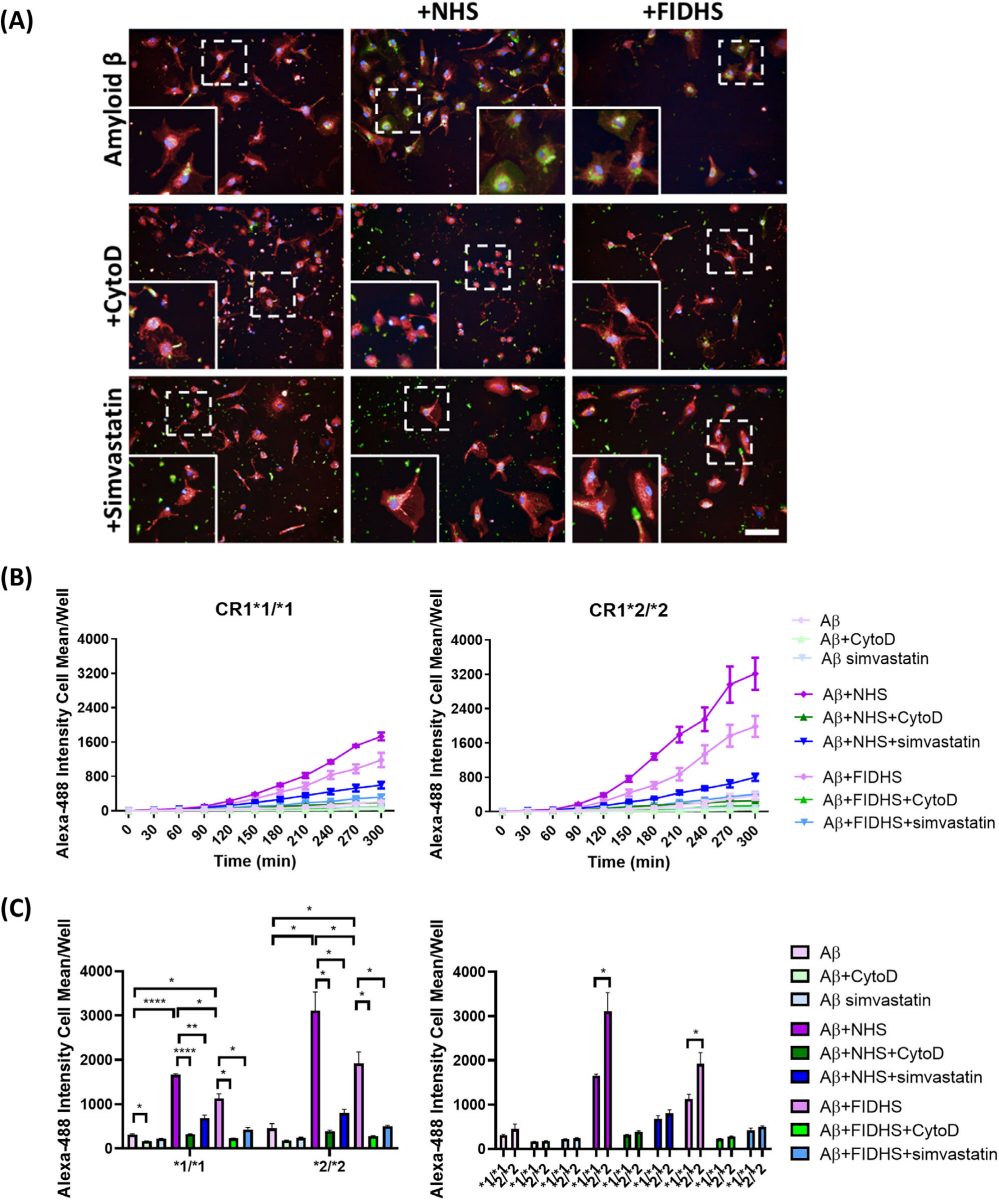
Induced pluripotent stem cell (iPSC) ‐microglia phagocytosis of HiLyte™ Fluor 488‐Beta‐Amyloid (1‐42). Phagocytic cargo was opsonized with normal human serum (NHS) or factor I (FI) ‐depleted human serum (FIDHS). Cytochalasin D (CytoD) was used as negative control. (A) Example pictures showing the cells at 2 h after phagocytosis (HiLyte™ Fluor 488‐Beta‐Amyloid: green; nuclei: blue; cell tracker: red). Scale bar: 100 µm. (B) Uptake in real time and (C) comparisons at 4 h of phagocytosis. iPSC‐microglia expressing risk variant of complement receptor 1 (CR1) display increased phagocytosis of HiLyte™ Fluor 488‐Beta—Amyloid (1 ‐ 42) when compared with non‐risk variant cells. Alexa‐488 Intensity cell mean describes the amount of HiLyte™ Fluor 488‐Beta‐Amyloid (1 ‐ 42) cellular fluorescence emission at each time point. Results are averages of the three independent iPSC lines per genotype, measured within a single 384‐well plate experiment. The phagocytosis assay was independently repeated at least three times demonstrating consistent results. *p < 0.05, **p < 0.01, and ****p < 0.0001.

Next, we tested phagocytosis of disease‐relevant cargos in real time. First, we tested uptake of Aβ aggregates, previously shown to be mediated by microglia in the AD brain.^24^ Fluorescent‐tagged Aβ aggregates were added to the cells and uptake recorded in real‐time; non‐opsonized Aβ aggregates were taken up slowly by all lines and uptake was ablated by CytoD; opsonization with NHS substantially increased the rate of uptake, while opsonization with FIDHS increased uptake compared to non‐opsonized, though significantly slower than the NHS‐opsonized group (Figure 3A,B). Phagocytic uptake of NHS‐opsonized Aβ aggregates was significantly faster in CR1*2‐expressing lines compared to CR1*1 expressors (Figure 3C). Uptake of FIDHS‐opsonized Aβ aggregates, much slower in both, remained significantly faster in CR1*2 expressing lines, while there was no significant difference between CR1*1‐ and CR1*2‐expressing lines in phagocytosis of non‐opsonized aggregates (Figure 3C). Addition of simvastatin significantly reduced phagocytosis of NHS‐opsonized Aβ aggregates.

**FIGURE 4:**
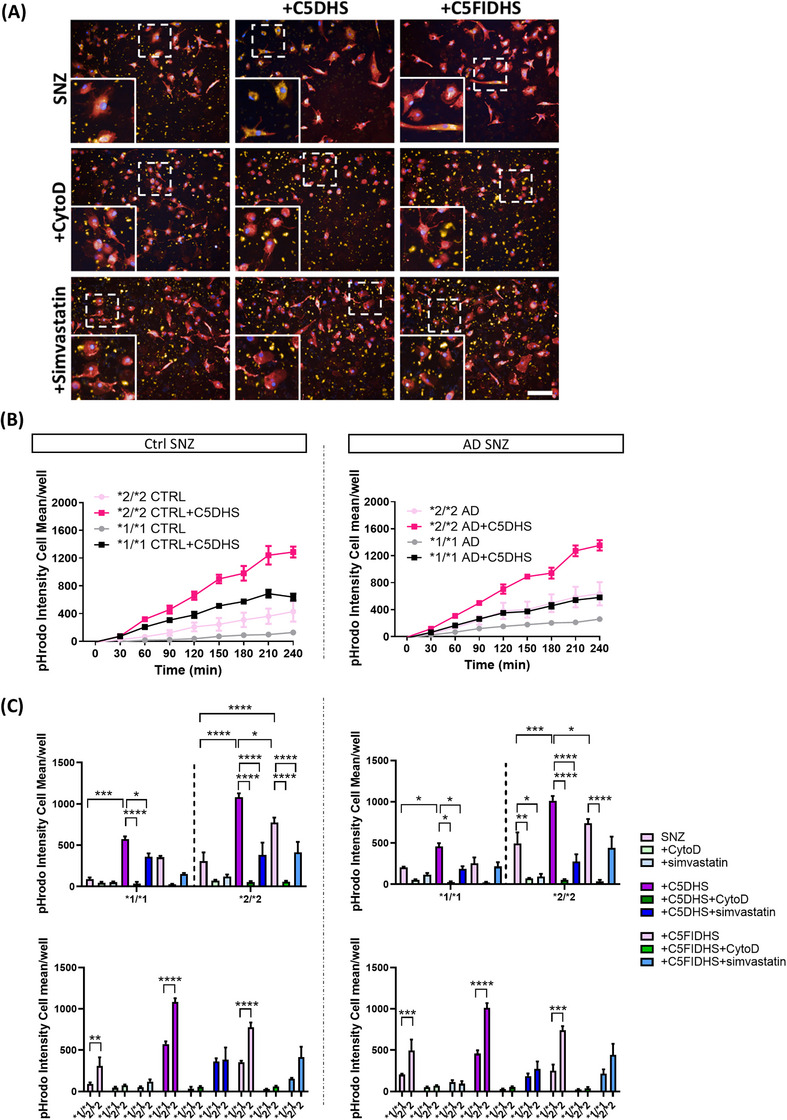
Induced pluripotent stem cell (iPSC) ‐microglia phagocytosis of pHrodo‐tagged human synaptoneurosomes (SNZ). Phagocytic cargo was opsonized with C5‐depleted human serum (C5DHS) or FI/C5‐depleted human serum (FIC5DHS). Cytochalasin D (CytoD) was used as negative control. (A) Example pictures showing the cells at 2 h after phagocytosis (pHrodo‐tagged SNZ: yellow; nuclei: blue; cell tracker: red). Scale bar: 100 µm. (B) Uptake in real time and (C) comparisons at 4 h of phagocytosis. iPSC‐microglia expressing risk variant of complement receptor 1 (CR1) display increased phagocytosis of human SNZ, control and AD, when compared with non‐risk variant expressing cells. pHrodo Intensity cell mean describes the amount of bioparticle cellular dye emission at each time point. Results are averages of the three independent iPSC lines per genotype, measured within a single 384‐well plate experiment. The phagocytosis assay was independently repeated at least three times demonstrating consistent results. *p < 0.05, **p < 0.01, ***p < 0.001, and ****p < 0.0001.

Finally, we tested phagocytosis of SNZ isolated from human brain, either from AD donors or healthy aged‐matched controls. Isolated SNZ were characterized by Western blotting, demonstrating enrichment of synaptic marker synaptophysin and reduction in Histone H3 compared to TBH (Figure S4), then labeled with pHrodo dye. In preliminary tests, exposure to NHS caused loss of labeled SNZ, likely a consequence of dye release caused by complement lysis; to circumvent this, C5DHS or FIC5DHS were used to opsonize. Non‐opsonized SNZ were taken up slowly by the cells and uptake was ablated by CytoD (Figure 4A,B). Rate of phagocytic uptake of SNZ opsonized by incubation with C5DHS was significantly increased, while uptake of FIC5DHS‐opsonized SNZ was much slower (Figure 4B,C). Cell lines expressing CR1*2 showed significantly faster phagocytosis of C5DHS‐opsonized SNZ compared to CR1*1 lines (Figure 4B,C). Uptake of FIC5DHS‐opsonized SNZ, much slower in both, remained significantly faster in CR1*2 expressing lines, while there was no significant difference between CR1*1‐ and CR1*2‐expressing lines in phagocytosis of non‐opsonized SNZ. No differences in phagocytic uptake were observed between SNZ isolated from control or AD human brain tissue. Addition of simvastatin significantly reduced phagocytosis of C5DHS‐opsonized SNZ isolated from control and AD brains.

**FIGURE 5:**
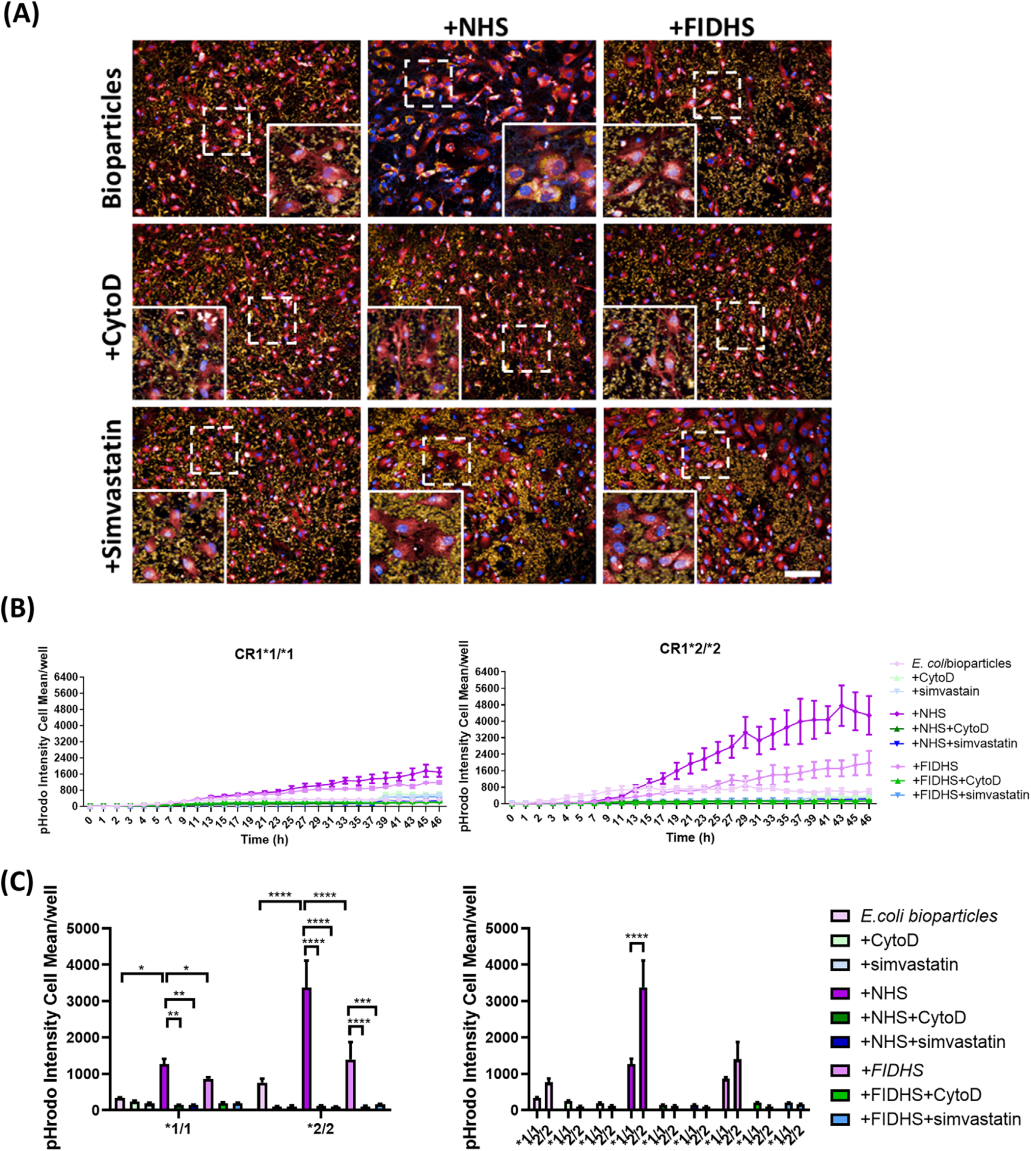
Induced pluripotent stem cell (iPSC) ‐astrocytes phagocytosis of pHrodo Red *E. coli* BioParticles. Phagocytic cargo was opsonized with normal human serum (NHS) or FI‐depleted human serum (FIDHS). Cytochalasin D (CytoD) was used as negative control. (A) Example pictures showing the cells at 24 h after phagocytosis (pHrodo‐tagged bioparticles: yellow; nuclei: blue; cell tracker: red). Scale bar: 100 µm. (B) Uptake in real time and (C) comparisons at 32 h of phagocytosis. iPSC‐astrocytes expressing risk variant of complement receptor 1 (CR1) display increased phagocytosis of pHrodo Red *E. coli* bioparticles when compared with non‐risk variant cells. pHrodo Intensity cell mean describes the amount of bioparticle cellular dye emission at each time point. Results are averages of the three independent iPSC lines per genotype, measured within a single 384‐well plate experiment. The phagocytosis assay was independently repeated at least three times demonstrating consistent results. *p < 0.05, **p < 0.01, and ****p < 0.0001.

**FIGURE 6:**
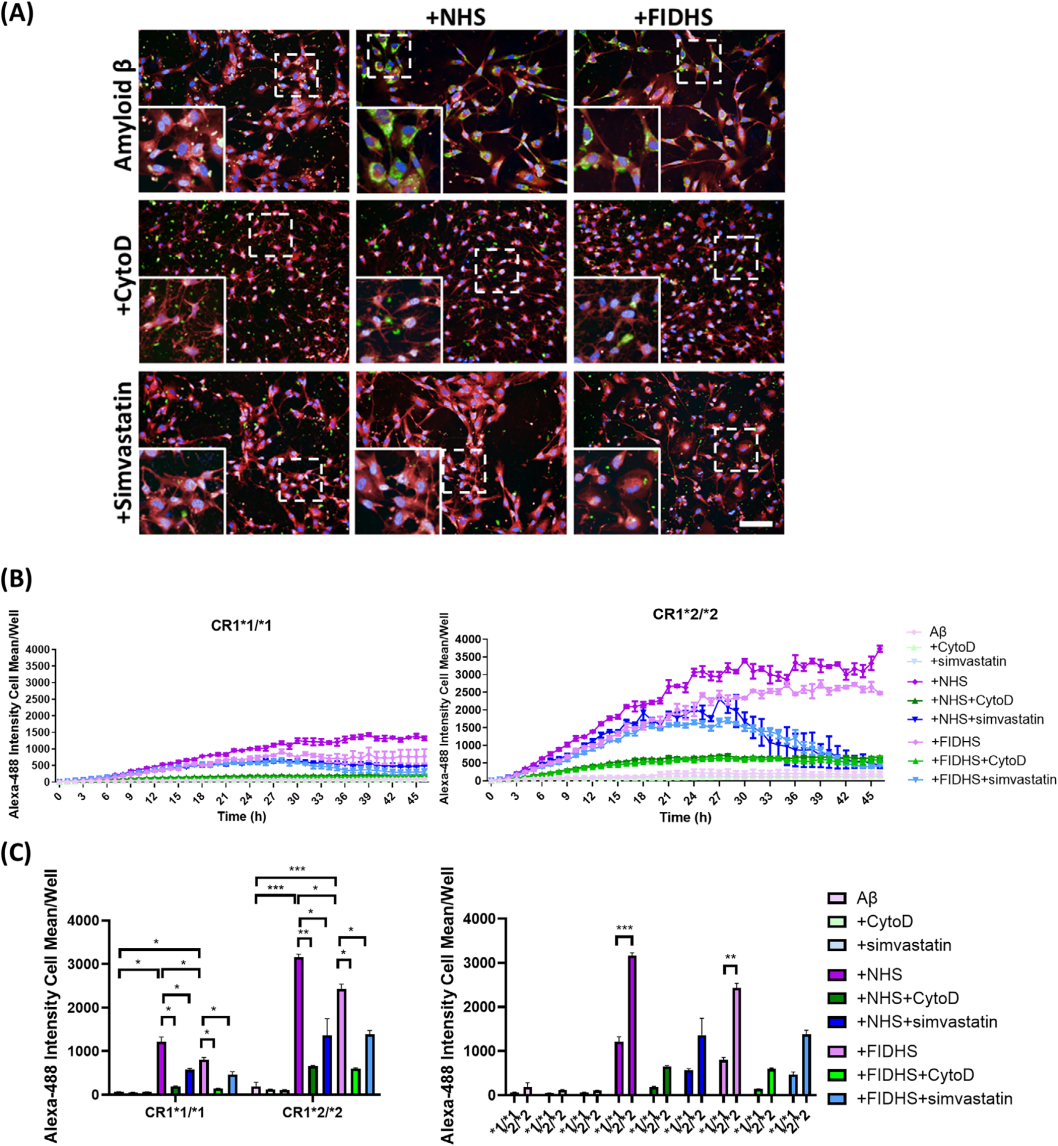
Induced pluripotent stem cell (iPSC) ‐astrocytes phagocytosis of HiLyte™ Fluor 488‐Beta‐Amyloid (1‐42). Phagocytic cargo was opsonized with normal human serum (NHS) or FI‐depleted human serum (FIDHS). Cytochalasin D (CytoD) was used as negative control. (A) Example pictures showing the cells at 24 h after phagocytosis (HiLyte™ Fluor 488‐Beta‐Amyloid: green; nuclei: blue; cell tracker: red). Scale bar: 100 µm. (B) Uptake in real time and (C) comparisons at 32 h of phagocytosis. iPSC‐astrocytes expressing risk variant of complement receptor 1 (CR1) display increased phagocytosis of HiLyte™ Fluor 488‐Beta‐Amyloid (1‐42) when compared with non‐risk variant cells. Prolonged incubation with simvastatin reduced astrocyte viability, leading to a decline in live phagocytosis, as depicted in the graph. Alexa‐488 Intensity cell mean describes the amount of HiLyte™ Fluor 488‐Beta‐Amyloid (1‐42) cellular fluorescence emission at each time point. Results are averages of the three independent iPSC lines per genotype, measured within a single 384‐well plate experiment. The phagocytosis assay was independently repeated at least three times demonstrating consistent results. *p < 0.05, **p < 0.01, and ***p < 0.001.

**FIGURE 7:**
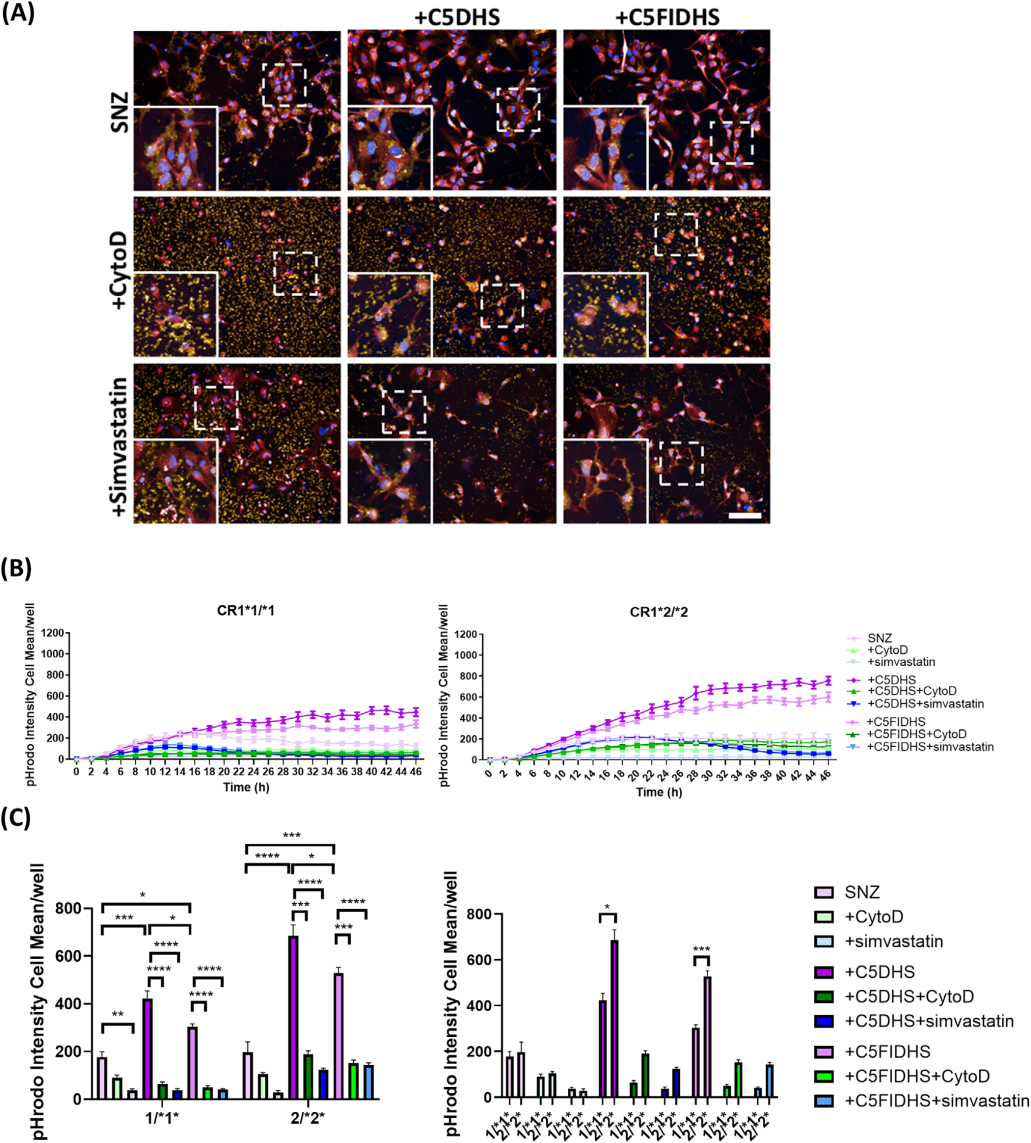
Induced pluripotent stem cell (iPSC) ‐astrocytes phagocytosis of pHrodo‐tagged human AD synaptoneurosomes (SNZ). Phagocytic cargo was opsonized with C5‐depleted human serum (C5DHS) or FI/C5‐depleted human serum (FIC5DHS). Cytochalasin D (CytoD) was used as negative control. (A) Example pictures showing the cells at 24 h after phagocytosis (pHrodo‐tagged SNZ: yellow; nuclei: blue; cell tracker: red). Scale bar: 100 µm. (B) Uptake in real time and (C) comparisons at 32 h of phagocytosis. iPSC‐astrocytes expressing risk variant of complement receptor 1 (CR1) display increased phagocytosis of human AD SNZ, when compared with non‐risk variant expressing cells. Prolonged incubation with simvastatin reduced astrocyte viability, leading to a decline in live phagocytosis, as depicted in the graph. pHrodo Intensity cell mean describes the amount of bioparticle cellular dye emission at each time point. Results are averages of the three independent iPSC lines per genotype, measured within a single 384‐well plate experiment. The phagocytosis assay was independently repeated at least three times demonstrating consistent results. **p < 0.05, **p < 0.01, ***p < 0.001, and ****p < 0.0001.

The above studies were replicated for iPSC astrocytes. Astrocytes showed phagocytic uptake for *E. coli* bioparticles, Aβ aggregates, and SNZ; uptake was much slower for astrocytes compared to microglia and was ablated by CytoD treatment (Figures 5, 6,7A). NHS opsonization markedly increased the rate of uptake for all targets, while FIDHS opsonization had a much reduced effect (Figure 5, 6,7B). Addition of simvastatin markedly reduced phagocytosis of NHS‐opsonized cargo and partially reduced uptake of non‐opsonized or FIDHS‐opsonized cargo. Comparison of iPSC‐astrocytes expressing the CR1*2 risk variant with those expressing the CR1*1 variant revealed a significantly faster rate of phagocytosis by all CR1*2 lines for all opsonized targets. Uptake of FIDHS‐opsonized targets, much slower for all, remained significantly faster in CR1*2 expressing lines, while there was no significant difference between CR1*1‐ and CR1*2‐expressing lines in phagocytosis of non‐opsonized aggregates (Figures 5, 6,7C).

## 4 DISCUSSION

There is now substantial evidence that complement plays a key role in AD pathogenesis.^25^, ^26^ GWAS in AD have linked complement genes, including *CR1* and *CLU*.^6^, ^13^ Emerging biomarker studies suggest that complement proteins in blood and cerebrospinal fluid may aid in distinguishing AD patients and predicting disease progression; however, further replication and standardization are needed before these markers can be considered reliable biomarkers for clinical use.^27^, ^28^ *Post mortem* analyses and studies in animal models show that complement components and activation products, notably C1q, C3b/iC3b, the proinflammatory activation products C3a and C5a and the terminal complement complex, are closely associated with AD pathology, and complement activation is implicated in amyloid clearance and synaptic loss.^29^, ^30^, ^31^, ^32^, ^33^, ^34^, ^35^, ^36^

Despite the strong genetic association of CR1 to AD risk, its precise function in the brain remains underexplored. We demonstrated CR1 expression in glial cells *ex vivo* and in healthy and AD brain, increased approximately five‐fold in the latter.^16^ In the periphery, CR1 plays key roles in opsonization and phagocytosis of complement‐coated particles, binding C3b fragments and catalyzing C3b cleavage to iC3b, the ligand for the phagocytic receptors CR3 and CR4 expressed on myeloid cells. We reasoned that CR1 likely played similar roles in the brain and that the AD risk and nonrisk variants differed in their capacity to facilitate phagocytosis. To test this, we generated iPSC lines homozygous for the AD non‐risk (CR1*1/CR1*1) and risk (CR1*2/CR1*2) variants, differentiated these into microglia and astrocytes, confirmed and quantified CR1 variant expression and tested phagocytic capacity for different cargos with or without cargo opsonization. Western blots of cell lysates from CR1*1‐expressing and CR1*2‐expressing microglia and astrocytes confirmed expression of the anticipated CR1 variant; quantification from these blots using a single‐site binding mAb revealed a five‐ to eight‐fold higher expression of CR1*1 compared to CR1*2 in microglia and astrocyte lines. In support of this finding, others reported that CR1 protein expression in brain was significantly reduced in samples from CR1*2‐expressing donors and suggested that this might predispose to complement dysregulation in brain.^37^

For all cargos and both cell types, NHS opsonization markedly increased the rate of phagocytic uptake, confirming the critical importance of complement‐mediated opsonization for efficient phagocytosis by glia, as previously demonstrated.^38^, ^39^ For both microglia and astrocytes, lines expressing the CR1*2 risk variant exhibited higher phagocytic activity compared to CR1*1 expressors for all cargos when opsonized but not when non‐opsonized, demonstrating a consistent functional difference between the variants. This was despite the lower levels of expression of CR1 on CR1*2 compared to CR1*1 expressing lines. This enhanced phagocytic activity may be attributed to the longer CR1*2 isoform facilitating better spatial cooperation with CR3 within the phagocytic machinery. To further assess whether the observed differences in phagocytosis related to altered capacity of CR1 variants to catalyze FI‐mediated C3b cleavage and generation of the CR3 ligand iC3b, cargos were opsonized with FIDHS. Flow cytometry of FIDHS‐opsonized phagocytic cargo confirmed absence of iC3b compared to the NHS‐opsonized group. For all cargos, phagocytosis after FIDHS‐opsonization, although reduced compared to NHS‐opsonized, remained much higher than for non‐opsonized, implying that CR1‐C3b interactions mediated phagocytosis without engagement of CR3, as recently observed by others for macrophage phagocytosis of bacteria.^40^ Notably, the rate of phagocytosis of FIDHS‐opsonized cargos was markedly reduced compared to NHS‐opsonized in both microglial and astrocyte lines; nevertheless, CR1*2‐expressing lines showed consistently faster uptake, suggesting either residual iC3b generation in FIDHS or an impact on phagocytosis even in the absence of iC3b generation. The observation that blocking CR3 using simvastatin almost completely abolished enhanced phagocytosis of NHS‐opsonized and FIDHS‐opsonized cargos supports the former suggestion; however, simvastatin also disrupts cholesterol synthesis, disrupting lipid rafts and clustering of phagocytic receptors, providing an alternative explanation for its major impact on phagocytosis in our studies.^41^, ^42^ Studies using alternative CR3 (and CR4) blockers are needed to clarify mechanism.

The demonstration that expression of the CR1 variant associated with AD risk enhances phagocytic activity of glia is counterintuitive. The authors of one of the earliest studies linking CR1*2 with AD risk speculated that the association was a consequence of increased number of C3b/C4b binding sites and resultant cofactor activity.^43^ Increased glial phagocytosis of opsonized amyloid and other debris would be expected to be beneficial by accelerating clearance; however, phagocytosis is a “double‐edged sword”, triggering cell damage and inflammation in some contexts.^44^, ^45^ Our findings suggest that glia expressing CR1*2 may act as “superphagocytes”, more efficient at clearing debris but also driving more inflammation and injury. In support of this concept, others have reported reduced brain Aβ levels in CR1*2 carriers implying enhanced amyloid clearance.^14^ An association of apolipoprotein E (APOE) ε4 expression with enhanced microglial phagocytosis compared to APOEε2 was also reported, another context where increased phagocytic activity is linked to expression of a disease risk variant.^46^

Our demonstration that astrocytes expressing CR1*2 also showed enhanced phagocytosis of opsonized targets, including synaptoneurosomes, may be particularly relevant in light of recent reports of a critical role for astrocytes in physiological and pathological synaptic elimination.^17^, ^47^, ^48^ Our findings imply that CR1*2‐expressing astrocytes may be more efficient eliminators of synapses, potentially enhancing neurodegeneration in AD; future work will assess the impact of expression of the CR1 variants on synaptic elimination by microglia and astrocytes utilizing in vitro and ex vivo models.

In conclusion, our findings highlight the importance of CR1 in the phagocytic activity of microglia and astrocytes and provide clues to the mechanism by which the CR1*2 variant incurs risk for AD. CR1 binds C3b on opsonized targets and catalyzes its cleavage to iC3b, the ligand for the phagocytic receptors CR3/4. Prevention of this cleavage event reduces but does not eliminate opsonization‐enhanced phagocytosis, implicating CR1 itself as a phagocytic receptor. On both microglia and astrocytes, the AD risk variant CR1*2 is associated with reduced protein expression yet paradoxically enhanced phagocytosis of complement‐opsonized cargos compared to CR1*1, an effect dependent on iC3b production and collaboration with CR3. Although this study provides numerous clues, the precise mechanism by which enhanced opsonic phagocytosis confers AD risk, and the impact of other AD risk variants in *CR1*, for example the coding variant in LHR‐D implicated in binding Αβ and C1q,^49^ remain to be elucidated. Understanding of mechanism may identify novel approaches to modulating phagocytosis for therapy in neurodegenerative disease.

## AUTHOR CONTRIBUTIONS

N.D. designed, led, and performed the experimental work and drafted the manuscript. B.P.M. conceived and supervised the project and finalized the manuscript. All authors contributed to aspects of the experimental work and read and approved the final manuscript. B.J.S. produced and provided the astrocytic conditioned media for terminal differentiation of induced pluripotent stem cells to microglia. W.M.Z. provided complement component depleted human serum and other key reagents and assays.

## CONFLICT OF INTEREST STATEMENT

B.P.M. serves as a consultant for Kira Pharmaceuticals, an advisory board member for Complement Therapeutics and a Director of Acionna Therapeutics. Author disclosures are available in the Supporting Information.

## CONSENT STATEMENT

Human subjects were involved in this research; informed consent was obtained.

## Supporting information



Supporting Information

Supporting Information

Supporting Information

Supporting Information

Supporting Information

Supporting Information

Supporting Information

## Data Availability

Information on the data underpinning the results presented here, including how to access them, can be found in the Cardiff University data catalogue at [DOI available on request from the authors].
