# Supplementary material for: The Alzheimer's disease‐associated complement receptor 1 variant confers risk by impacting glial phagocytosis
Source: Alzheimers Dement. 2025 Jul 9;21(7):e70458. doi: 10.1002/alz.70458 (PMC12238831; doi:10.1002/alz.70458)
Supplement: Supplementary file 2 — Supporting Information [file ALZ-21-e70458-s005.docx]

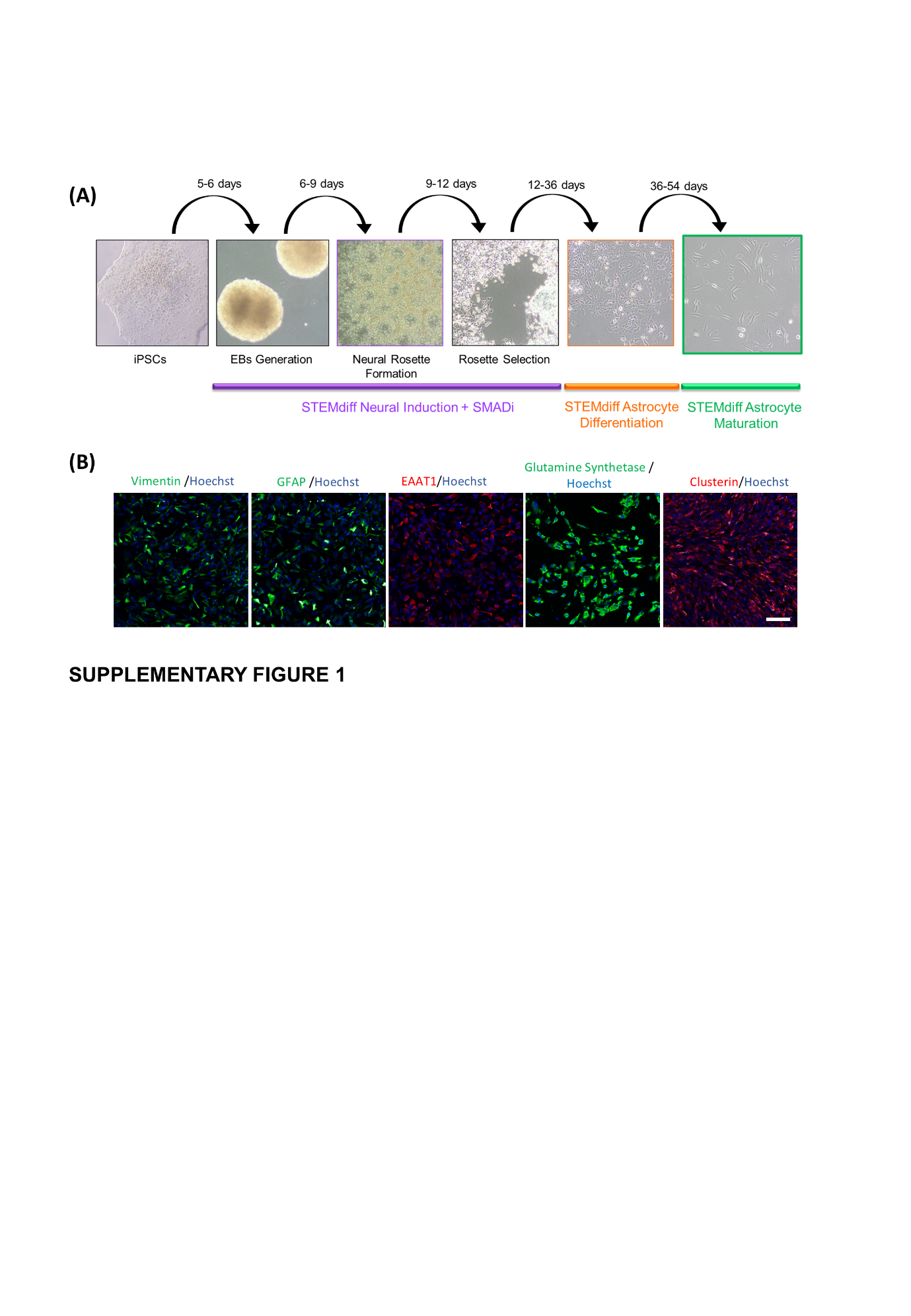


**SUPPLEMENTARY FIGURE 1.** **Differentiation of donor‐derived iPSC to astrocytes and characterization. (A)** Procedure of the differentiation of iPSC to iPSC‐astrocytes using the STEMdiff™ Astrocyte Differentiation Kit and the STEMdiff™ Astrocyte Maturation Kit using the manufacturer’s EB protocol (STEMCELL Technologies). **(B)** Characterization of astrocytes for the expression of vimentin, GFAP, glutamine synthetase as well as clusterin. Scale bar: 100 μm.
