# Supplementary material for: The Alzheimer's disease‐associated complement receptor 1 variant confers risk by impacting glial phagocytosis
Source: Alzheimers Dement. 2025 Jul 9;21(7):e70458. doi: 10.1002/alz.70458 (PMC12238831; doi:10.1002/alz.70458)
Supplement: Supplementary file 3 — Supporting Information [file ALZ-21-e70458-s004.docx]

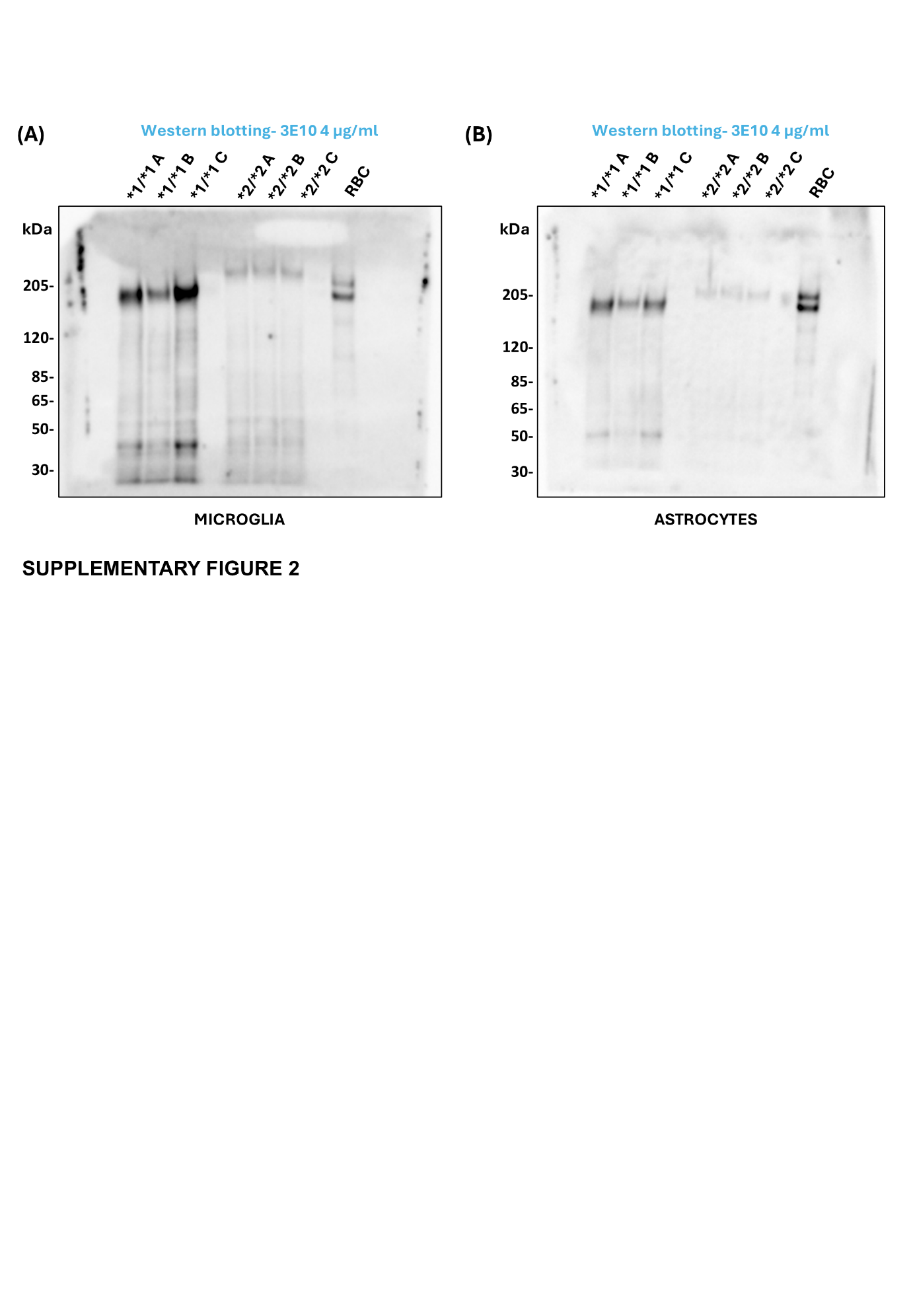
**SUPPLEMENTARY FIGURE 2.** Uncropped western blots showing staining of **(A)** microglia and **(B)** astrocytes using the 3E10 mAb against CR1.
