# Supplementary material for: The Alzheimer's disease‐associated complement receptor 1 variant confers risk by impacting glial phagocytosis
Source: Alzheimers Dement. 2025 Jul 9;21(7):e70458. doi: 10.1002/alz.70458 (PMC12238831; doi:10.1002/alz.70458)
Supplement: Supplementary file 4 — Supporting Information [file ALZ-21-e70458-s002.docx]

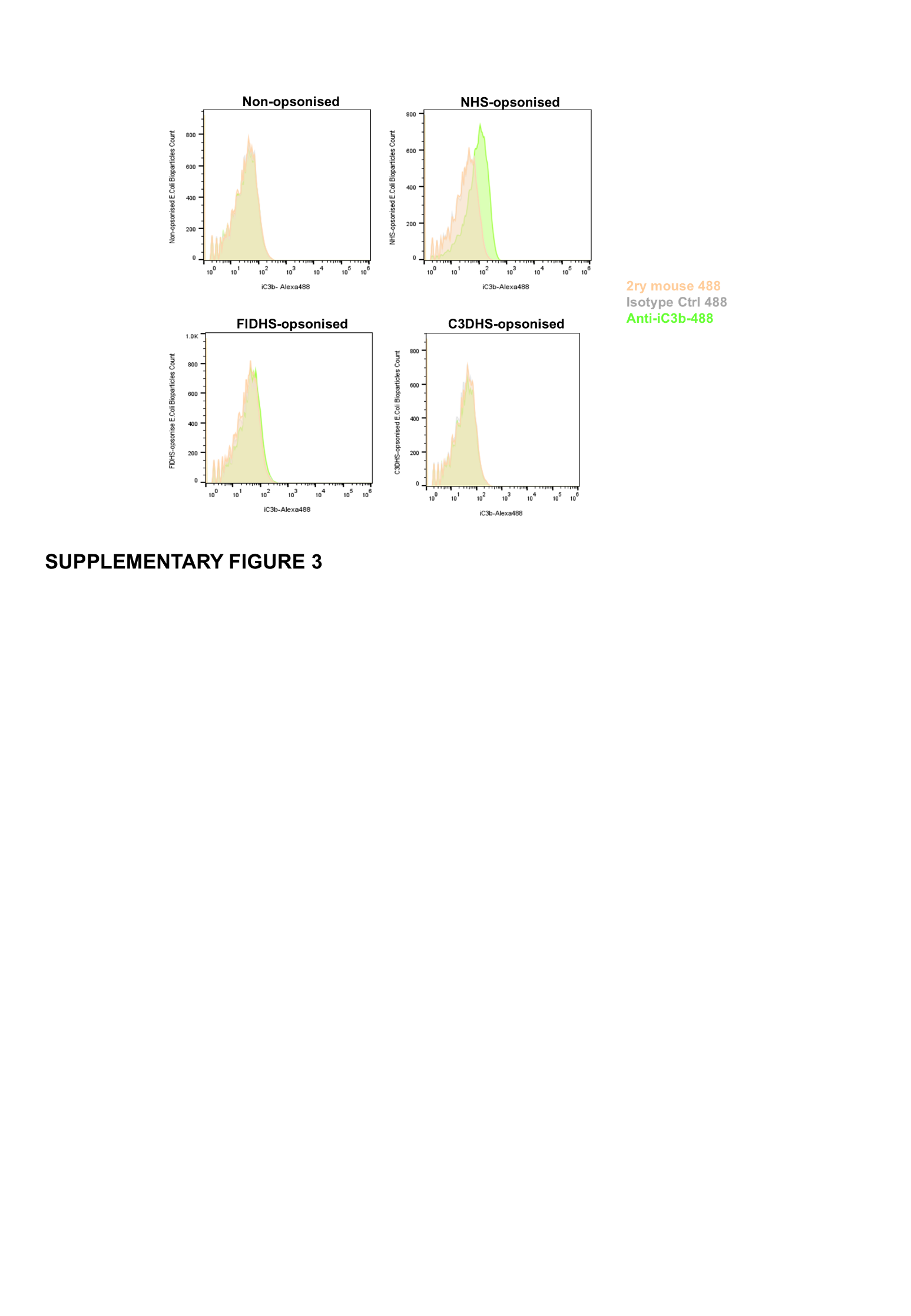


**SUPPLEMENTARY FIGURE 3. iC3b opsonization of pHrodo™ Red *E.coli* BioParticles™.** Flow cytometry analysis showing iC3b opsonization of bioparticles using an anti-iC3b antibody (Quidel A290) after incubation with normal human serum (NHS). iC3b was not detected on bioparticles opsonized with FI-depleted or C3-depleted human serum (FIDHS or C3DHS), nor on non-opsonized bioparticles. Isotype and primary-only controls tested negative.
