# Supplementary material for: The Alzheimer's disease‐associated complement receptor 1 variant confers risk by impacting glial phagocytosis
Source: Alzheimers Dement. 2025 Jul 9;21(7):e70458. doi: 10.1002/alz.70458 (PMC12238831; doi:10.1002/alz.70458)
Supplement: Supplementary file 5 — Supporting Information [file ALZ-21-e70458-s006.docx]

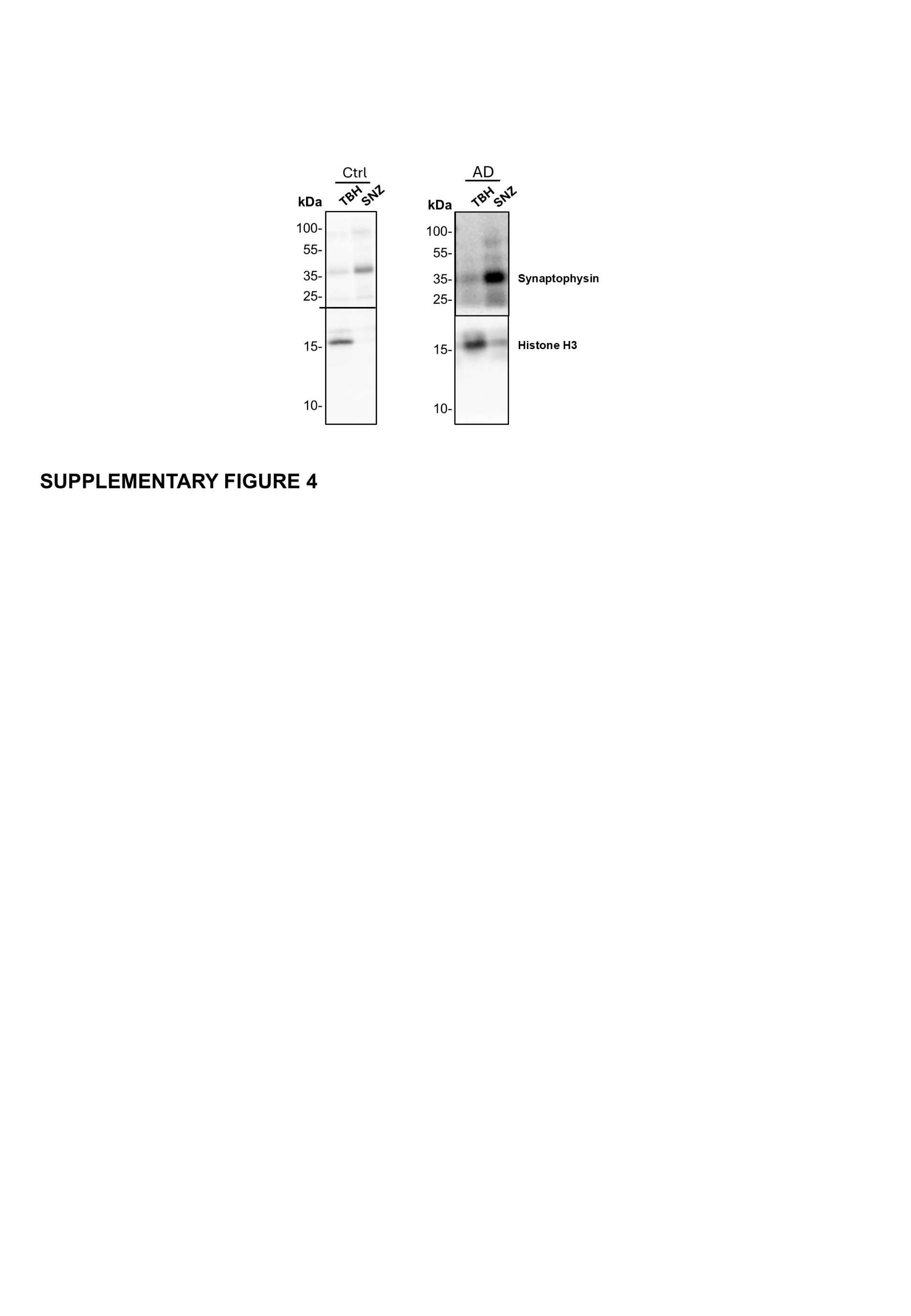


**SUPPLEMENTARY FIGURE 4.** **Synaptoneurosome isolation confirmation via western blotting.** Western blots displaying detection of synaptic marker synaptophysin (1:500, rabbit mAb, Abcam ab32127) and Histone H3 (1:1000, rabbit pAb, Abcam ab1791) in human total brain homogenate (TBH) and synaptoneurosomes (SNZ) (25 µg/lane) from one control and on AD human brain sample used for phagocytosis. Synaptophysin is enriched and histone H3 depleted in SNZ samples compared to TBH. The control lane blot is part of a supplementary figure used by our group for another publication ^1^, Hu 3 lane in figure S8, because the control synaptoneurosomes were used in multiple studies.

1. Byrne RAJ, Nimmo J, Torvell M, et al. The schizophrenia-associated gene CSMD1 encodes a complement classical pathway inhibitor predominantly expressed by astrocytes and at synapses in mice and humans. *Brain Behav Immun*. 2025;127:287-302. doi:10.1016/j.bbi.2025.03.026
