# Supplementary material for: The Alzheimer's disease‐associated complement receptor 1 variant confers risk by impacting glial phagocytosis
Source: Alzheimers Dement. 2025 Jul 9;21(7):e70458. doi: 10.1002/alz.70458 (PMC12238831; doi:10.1002/alz.70458)
Supplement: Supplementary file 6 — Supporting Information [file ALZ-21-e70458-s003.docx]

**SUPPLEMENTARY TABLE 1** Human brain tissue information. BBN is Brain Bank Network ID.

| **ID** | **BNN** | **AD/ control** | **Sex** | **Age (years)** |
| --- | --- | --- | --- | --- |
| **SD040/21** | BBN001.36924 | Braak VI | F | 72 |
| **SD030/21** | BBN001.36839 | Braak VI | M | 75 |
| **SD015/21** | BBN001.36689 | Braak VI | M | 75 |
| **SD012/21** | BBN 001.36433 | Braak VI | M | 78 |
| **SD027/20** | BBN001.36346 | Braak VI | F | 90 |
| **SD042/18** | BBN001.35138 | Control | F | 73 |
| **SD046/17** | BBN 001.31504 | Control | F | 65 |
| **SD024/17** | BBN001.30178 | Control | M | 72 |
| **SD012/17** | BBN001.29882 | Control | F | 71 |
| **SD030/18** | BBN001.34150 | Control | M | 63 |
