# Supplementary material for: The Alzheimer's disease‐associated complement receptor 1 variant confers risk by impacting glial phagocytosis
Source: Alzheimers Dement. 2025 Jul 9;21(7):e70458. doi: 10.1002/alz.70458 (PMC12238831; doi:10.1002/alz.70458)
Supplement: Supplementary file 7 — Supporting Information [file ALZ-21-e70458-s001.docx]

**SUPPLEMENTARY TABLE 2** Characteristics of donor-derived iPSC lines

| **iPSC Line ID** | **Sex** | **Clinical Details** | **CR1 length polymorphism** | **CR1 density polymorphism** | **APOE Status** |
| --- | --- | --- | --- | --- | --- |
| B1-5 | M | healthy | *1/*1 | H/H | ε3/ε3 |
| B2-4 | F | healthy | *1/*1 | H/L | ε3/ε3 |
| B3-10 | M | healthy | *1/*1 | H/H | ε3/ε4 |
| A1 | M | healthy | *2/*2 | H/H | ε3/ε3 |
| A3 | M | healthy | *2/*2 | H/H | ε3/ε3 |
| D7 | F | healthy | *2/*2 | ND | ε2/ε4 |
